# Rational Engineering of Enzyme Allosteric Regulation through Sequence Evolution Analysis

**DOI:** 10.1371/journal.pcbi.1002612

**Published:** 2012-07-12

**Authors:** Jae-Seong Yang, Sang Woo Seo, Sungho Jang, Gyoo Yeol Jung, Sanguk Kim

**Affiliations:** 1 School of Interdisciplinary Bioscience and Bioengineering, Pohang University of Science and Technology, Pohang, Gyeongbuk, Korea; 2 Department of Chemical Engineering, Pohang University of Science and Technology, Pohang, Gyeongbuk, Korea; 3 Division of Molecular and Life Science, Pohang University of Science and Technology, Pohang, Gyeongbuk, Korea; 4 Division of IT Convergence Engineering, Pohang University of Science and Technology, Pohang, Gyeongbuk, Korea; MRC Laboratory of Molecular Biology, United Kingdom

## Abstract

Control of enzyme allosteric regulation is required to drive metabolic flux toward desired levels. Although the three-dimensional (3D) structures of many enzyme-ligand complexes are available, it is still difficult to rationally engineer an allosterically regulatable enzyme without decreasing its catalytic activity. Here, we describe an effective strategy to deregulate the allosteric inhibition of enzymes based on the molecular evolution and physicochemical characteristics of allosteric ligand-binding sites. We found that allosteric sites are evolutionarily variable and comprised of more hydrophobic residues than catalytic sites. We applied our findings to design mutations in selected target residues that deregulate the allosteric activity of fructose-1,6-bisphosphatase (FBPase). Specifically, charged amino acids at less conserved positions were substituted with hydrophobic or neutral amino acids with similar sizes. The engineered proteins successfully diminished the allosteric inhibition of *E. coli* FBPase without affecting its catalytic efficiency. We expect that our method will aid the rational design of enzyme allosteric regulation strategies and facilitate the control of metabolic flux.

## Author Summary

Design of allosterically regulatable enzyme is essential to develop a highly efficient metabolite production. However, mutations on allosteric ligand binding sites often disrupt the catalytic activity of enzyme. To aid the design process of allosterically controllable enzymes, we develop an effective computational strategy to deregulate the allosteric inhibition of enzymes based on sequence evolution analysis of allosteric ligand-binding sites. We analyzed the molecular evolution and amino acid composition of catalytic and allosteric sites of enzymes, and discovered that allosteric sites are evolutionarily variable and comprised of more hydrophobic residues than catalytic sites. We then experimentally tested our strategy of enzyme allosteric regulation and found that the designed mutations effectively deregulated allosteric inhibition of FBPase. We believe that our method will aid the rational design of enzyme allosteric regulation and help to facilitate control of metabolic flux.

## Introduction

Living cells coordinate metabolic flux through the allosteric regulation of enzymatic activity [1]–[3]. Allosteric sites provide a molecular platform for allosteric regulators, which are spatially apart from but energetically coupled with catalytic sites [4], [5]. Binding of allosteric regulators induces an interaction rearrangement of allosteric residues and regulates enzymatic activity [6]. One of the challenges of rational allosteric control is to design mutants that do not impair catalytic function but change binding specificity to allosteric regulators. In the emerging era of engineering enzymatic substrates through active-site remodeling [7]–[9], deregulating allosteric inhibition is necessary for removing allosteric behavior of template enzymes to obtain commodities. For example, the productivity of ethanol fermentation process is dramatically increased by a mutation in pyruvate dehydrogenase complex which leads to the complex being less sensitive to the allosteric inhibition by NADH [10]. Also, lysine production is increased by deregulation of the allosteric inhibition of aspartokinase [11]. Structures of enzyme-ligand complexes have provided the molecular details of enzymatic regulation; however, the underlying principles of allosteric regulation still need to be uncovered in order to engineer allosterically controllable enzymes.

Allosteric and catalytic sites are similar in the sense that they both bind to specific ligands, but have been exposed to different evolutionary constraints. For example, the allosteric sites of mammalian phosphofructokinase were arisen from gene duplication and fusion, and differ across orthologues [12]. Also, the inhibitor binding site of glycogen phosphorylases has changed over the course of evolution from yeast to vertebrates, while the residues of catalytic sites are conserved [13]. Catalytic sites are responsible for substrate binding and conversion, thus mutations in catalytic sites usually demolish the catalytic function of enzymes [14]. Therefore, catalytic sites are usually highly conserved [15], [16]. On the other hand, allosteric sites provide binding platforms for ligands but are not involved in the catalytic conversion of ligands. Moreover, allosteric regulation mechanisms often varied across species living in different environments at organism level, suggesting that residues in allosteric sites have evolved to adapt to their environments. For instance, adenosine monophosphate (AMP) can synergistically inhibit porcine FBPase with fructose 2,6-bisphosphate, but this synergism has not been found in *E. coli* FBPase [17]. Therefore, amino acid residues in allosteric sites may be subject to change to control the allosteric behavior of enzymes in different species.

In this study, we systematically analyzed the molecular evolution of enzyme allosteric sites and discovered that allosteric sites have evolved along different evolutionary pathways compared to the highly conserved catalytic sites. We also compared the amino acid compositions of catalytic and allosteric sites, and discovered that allosteric sites have lower numbers of charged residues than do catalytic sites. We then introduced mutations into the allosteric sites of *E. coli* FBPase to control allosteric regulation without impairing catalytic activity. We confirmed that even at high doses of allosteric inhibitors, mutant *E. coli* FBPase maintained its catalytic activity. Understanding the evolutionary basis of enzyme allosteric control will provide a method to efficiently engineer tailor-made enzymes to control allosteric regulation.

## Results

### Allosteric sites are less conserved than catalytic sites

**Figure 1:**
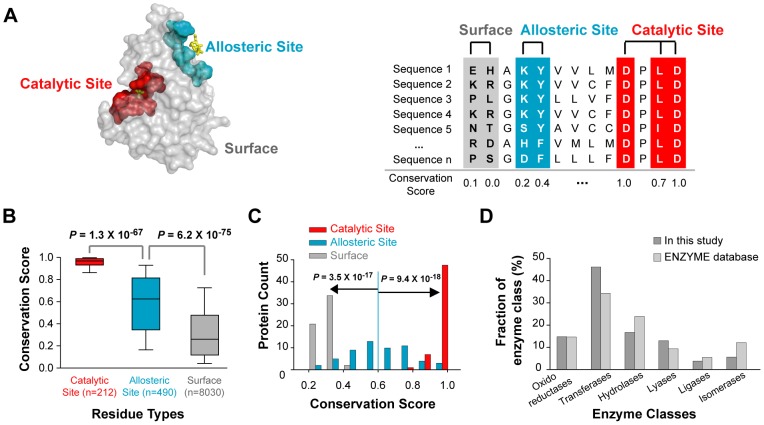
Investigation of conservation scores from catalytic and allosteric sites of enzymes. (A) Representative mapping of catalytic, allosteric, and surface residues on 3D structures, represented in red, cyan, and gray, respectively. Allosteric sites of enzymes are generally located away from catalytic sites. Conservation scores were calculated for each residue from homologous sequences collected from the UniProtKB/SwissProt database. To compare conservation scores among different proteins, we applied the percentile normalization method. Conservation scores range from 0 to 1. Highly conserved residues get larger conservation scores. (B) Distributions of conservation scores for catalytic, allosteric, and surface residues. Shown are the distributions of conservation scores of residues collected from 56 allosteric proteins. The annotation of each residue comes from hand-curated databases. (C) Distributions of average conservation scores of catalytic, allosteric, and surface residues per protein. (D) Distributions of enzyme classes in our dataset and in the entire ENZYME database. The statistical significance (*P*-value) was measured by the Mann-Whitney U test.

To understand the differences in the evolutionary properties of catalytic and allosteric sites, we investigated sequence conservation of catalytic and allosteric sites in 56 enzyme structures. Each enzyme has single catalytic and allosteric sites, which are composed of total 212 and 490 residues respectively. The annotations for catalytic and allosteric residues were obtained from databases, experimentally determined enzyme-ligand complex structures, and biochemical studies [18], [19]. Sequence conservation scores were calculated from multiple sequence alignments of homologous sequences collected from various species (Fig. 1*A*, see Materials and Methods for details). As shown in Fig. 1*B*, residues of allosteric sites (average conservation score = 0.58) are significantly less conserved than are residues of catalytic sites (average conservation score = 0.94, *P* = 1.3×10^−67^, Table S1 *A*), although both sites are significantly more conserved than the rest of the surface (*P* = 6.2×10^−75^). To confirm that these differences in evolutionary conservation were true for each protein sequence, we analyzed the average conservation score distributions of allosteric and catalytic sites and confirmed that the distributions were significantly different (*P* = 9.4×10^−18^, Fig. 1*C*, Table S1 *B*). Furthermore, as shown in Fig. 1*D*, our observation is not biased toward certain enzyme commission classes (Table S2). This indicates that evolutionary constraints are significantly different on allosteric and catalytic sites.

We also tested our analysis on different criteria for selecting 522 catalytic and 782 allosteric site residues based on the 56 enzyme-ligand complex structures to confirm our observation. Catalytic and allosteric residues were selected as those within 6 Å of the respective ligands/substrates in the complex structures and compared with the annotation of catalytic and allosteric sites in databases (Table S1 *C*, see Materials and Methods for details). We found that sequence conservation patterns of catalytic and allosteric residues selected from structures were similar to those of residues selected from the annotated database. Specifically, allosteric sites (average conservation score = 0.58) were significantly less conserved than catalytic sites (average conservation score = 0.82, *P* = 3.2×10^−58^, Fig. S1 *A*, Table S1 *C*). As before, we confirmed that conservation ratios between catalytic and allosteric sites in enzyme structures were significantly different in individual proteins (Text S1). Interestingly, allosteric sites were found to have a broader range of conservation scores compared to catalytic sites (*P* = 1.0×10^−61^, *F*-test, Fig. 1*B*).

Allosteric sites are composed of evolutionarily more variable residues than catalytic sites, even though these interact with ligands just as in the case of catalytic sites. We were intrigued by these observations because ligand-binding sites are generally known to be conserved across species [16], [20], [21]. We speculated that the naturally variable residues in allosteric sites might constitute potential targets for engineering the allosteric regulation of enzymes without impairing their catalytic activities.

### Allosteric sites are more hydrophobic than catalytic sites

**Figure 2:**
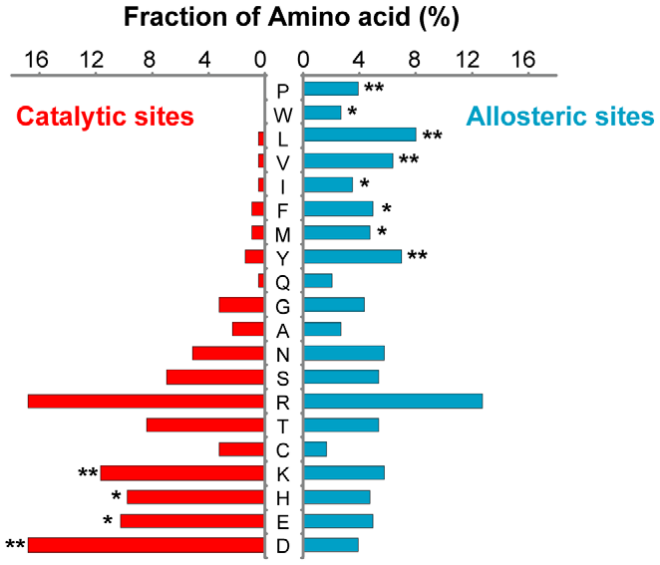
Amino acid compositions of catalytic and allosteric residues. The fraction of each amino acid of catalytic and allosteric residues is shown. Allosteric sites have more hydrophobic residues compared to catalytic sites, while catalytic sites have more charged amino acids than do allosteric sites. The statistical significance (*P*-value) was measured by Fisher's exact test; *P<0.05 and **P<0.005.

Next, we investigated the physicochemical properties of the catalytic and allosteric sites of 56 enzymes by comparing their amino acid compositions. We found that charged residues such as lysine, histidine, glutamic acid, and aspartic acid were highly enriched in catalytic sites (*P* = 5.5×10^−14^, Fig. 2, Table S3 *A*), whereas hydrophobic residues such as proline, tryptophan, leucine, valine, isoleucine, phenylalanine, methionine, and tyrosine were highly enriched in allosteric sites (*P* = 2.9×10^−26^, Fig. 2, Table S3 *A*). We confirmed these observations with a different set of catalytic and allosteric sites derived from enzyme-ligand complex structures (Fig. S1 *B*, Table S3 *B*). These differences in amino acid composition could be due to the different functional roles of the sites. In catalytic sites, ligands are subject to the heterolytic breakage and formation of covalent bonds, but such bond breakage and formation do not occur in allosteric sites. Hydrophilic residues often participate in a hydrogen-bonded network within the active site to facilitate bond breakage or formation [22], [23]. On the other hand, hydrophobic residues are enriched in allosteric sites to provide a binding pocket for the ligand, with only a small fraction of charged residues that are present to facilitate specific interactions during ligand binding. Thus, we postulated that the allosteric behavior of the enzyme can be changed by mutating the evolutionarily variable residues in allosteric sites.

### Sequence evolution of the allosteric sites in FBPase

**Figure 3:**
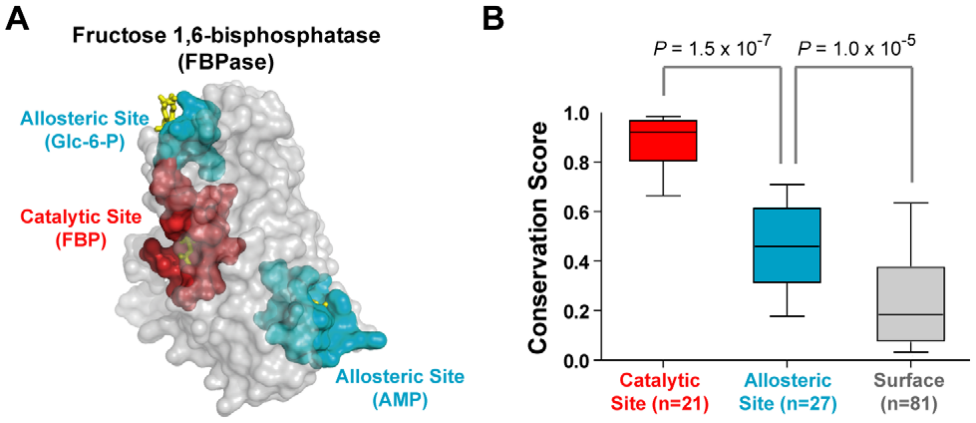
Comparison of evolutionary properties of catalytic and allosteric site residues of fructose 1,6-bisphosphatase (FBPase). (A) Structure of the *E.coli* FBPase (PDB code 2Q8M). Catalytic site (21 residues) and allosteric site (27 residues) residues were defined as amino acid residues within 6 Å of the substrate, and represented in red and cyan colors, respectively. (B) Distributions of conservation scores for catalytic, allosteric, and surface residues. The statistical significance (*P*-value) was measured by the Mann-Whitney U test.

We chose FBPase as a model system in which to test our hypothesis of allosteric site evolution. FBPase is a key metabolic enzyme in the gluconeogenic pathway, and has one catalytic site and two distinct allosteric sites that provide binding platforms for AMP and glucose-6-phosphate (Glc-6-P, Fig. 3*A*) [17]. Activation of the gluconeogenic pathway changes the carbon flux toward the pentose phosphate pathway and increases the level of NADPH that can be utilized to produce various desirable metabolites such as amino acids, fatty acids, and hydrogen [24]–[26]. To activate the gluconeogenic pathway under high glucose concentrations, the allosteric inhibition of FBPase by both AMP and Glc-6-P should be eliminated while maintaining its catalytic activity.

We examined the sequence evolution of FBPase and found that the allosteric sites were significantly less conserved than the catalytic site (*P* = 1.5×10^−7^, Fig. 3*B*), but more conserved than rest of the surface residues (*P* = 1.0×10^−5^, Fig. 3*B*). Based on the enzyme-ligand complex structure, we selected residues from the catalytic (21 residues) and allosteric (27 residues) sites that were within 6 Å of the substrates, fructose-1,6-bisphosphate (FBP), and the allosteric inhibitors (AMP and Glc-6-P, Table S4). The rest of the surface area was selected from solvent accessible residues of the enzyme.

### Designed mutations diminished the allosteric regulation of FBPase

**Figure 4:**
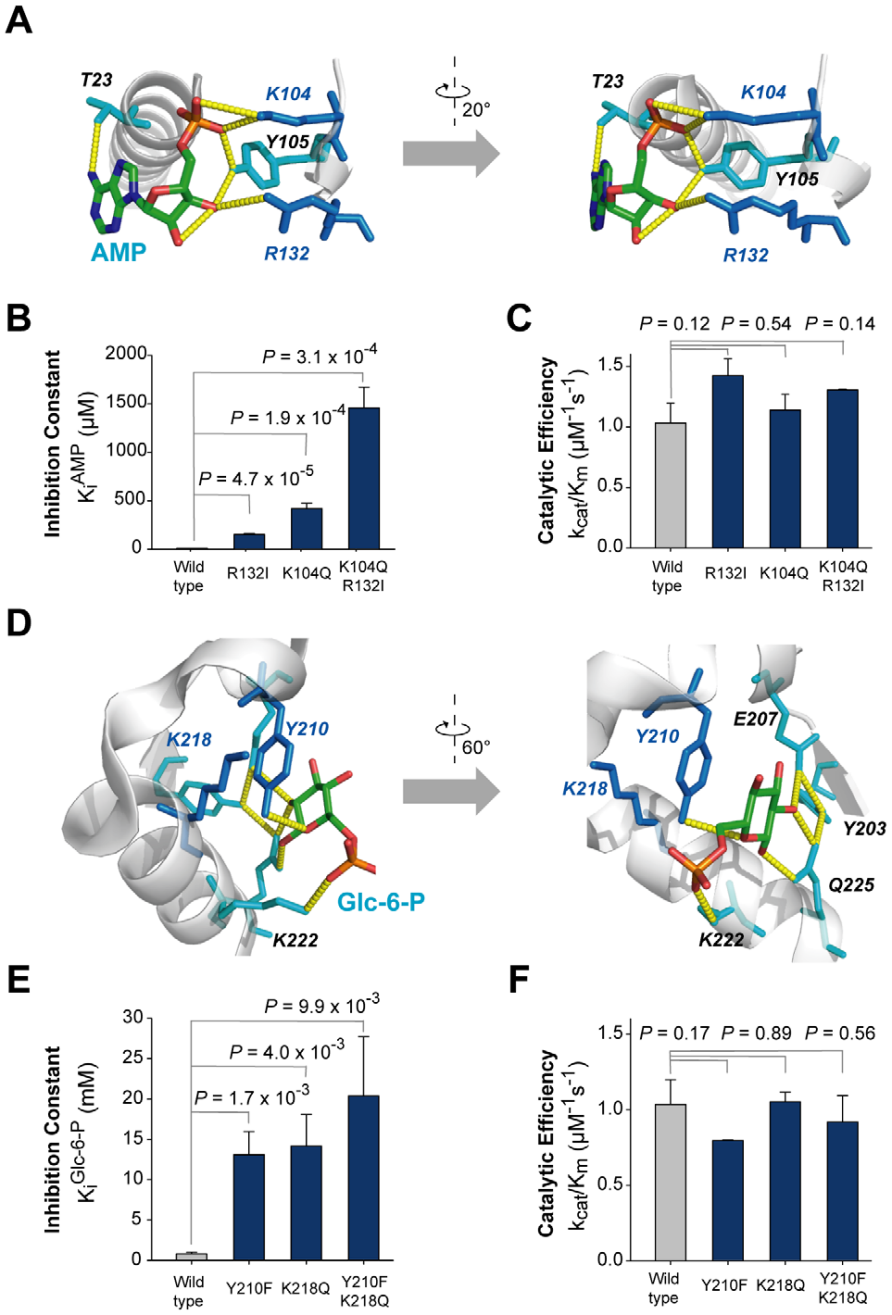
Mutations in each allosteric site of *E. coli* FBPase. (A) Residues in the allosteric regulator AMP binding site. Four residues T23, K104, Y105, and R132 have hydrogen bonds and/or polar contacts, represented by yellow dotted lines, with AMP. The mutated positions are shown in blue and cyan. In particular, less conserved residues are shown in blue. (B) Comparison of inhibition constants of wild-type and mutant FBPase by the allosteric inhibitor, AMP. The statistical significance (*P*-value) was measured by t-test. (C) Comparison of the catalytic efficiencies of wild-type FBPase and AMP binding site mutants. (D) Residues in the allosteric regulator Glc-6-P binding site. Five residues Y203, E207, Y210, K222, and Q225 have hydrogen bonds and/or polar contacts, represented by yellow dotted lines, with Glc-6-P; K218 interlocks with Y210. The mutated positions are shown in blue and cyan. In particular, less conserved residues are shown in blue. (E) Comparison of inhibition constant of wild-type and mutant FBPase by the allosteric inhibitor, Glc-6-P. (F) Comparison of catalytic efficiencies of wild-type FBPase and Glc-6-P binding site mutants.

To alleviate the allosteric regulation of FBPase, we selected residues that have favorable binding interactions with AMP or Glc-6-P (Fig. 4*A and D*). For the inhibitory effect of AMP with respect to FBP, less conserved and charged residues (R132, K104) were mutated (Fig. 4*A*, Text S2). As shown in Fig. 4*B*, the single mutants R132I and K104Q showed 15-fold (*P* = 4.7×10^−5^) and 40-fold (*P* = 1.9×10^−4^) higher inhibition constants (*K_i_*), respectively, than the wild type (Table S5). As both residues are directly involved in electrostatic interactions with AMP, each single mutant that diminished the interaction showed only modest effects on the binding of the anionic allosteric effector. However, in the case of the double mutant (K104Q/R132I), the *K_i_* was 140-fold (*P* = 3.1×10^−4^) higher compared to the wild type, indicating that these mutations had a synergistic effect on disturbing the binding of AMP. We confirmed that mutations in the AMP binding pocket did not affect the regulatory control of Glc-6-P (Fig. S2 *B*). Next, the less conserved residues Y210 and K218 in the Glc-6-P binding pocket were also mutated (Fig. 4*D*, Text S3). Both the Y210F (*P* = 1.7×10^−3^) and K218Q (*P* = 4.0×10^−3^) mutants had about 17-fold higher *K_i_* values than the wild type (Fig. 4*E*). In case of the double mutant (Y210F/K218Q), the *K_i_* value was 25-fold higher than the wild type (*P* = 9.9×10^−3^, Fig. 4*E*). Mutations in the Glc-6-P binding pocket did not affect the regulatory properties of AMP to FBPase (Fig. S2 *C*). Notably, the catalytic efficiency (*k_cat_*/*K_m_*) of each mutant was sustained or slightly increased than that of wild-type FBPase, although allosteric regulation by AMP and Glc-6-P was perturbed (*P*>0.1, Fig. 4*C and F*).

**Figure 5:**
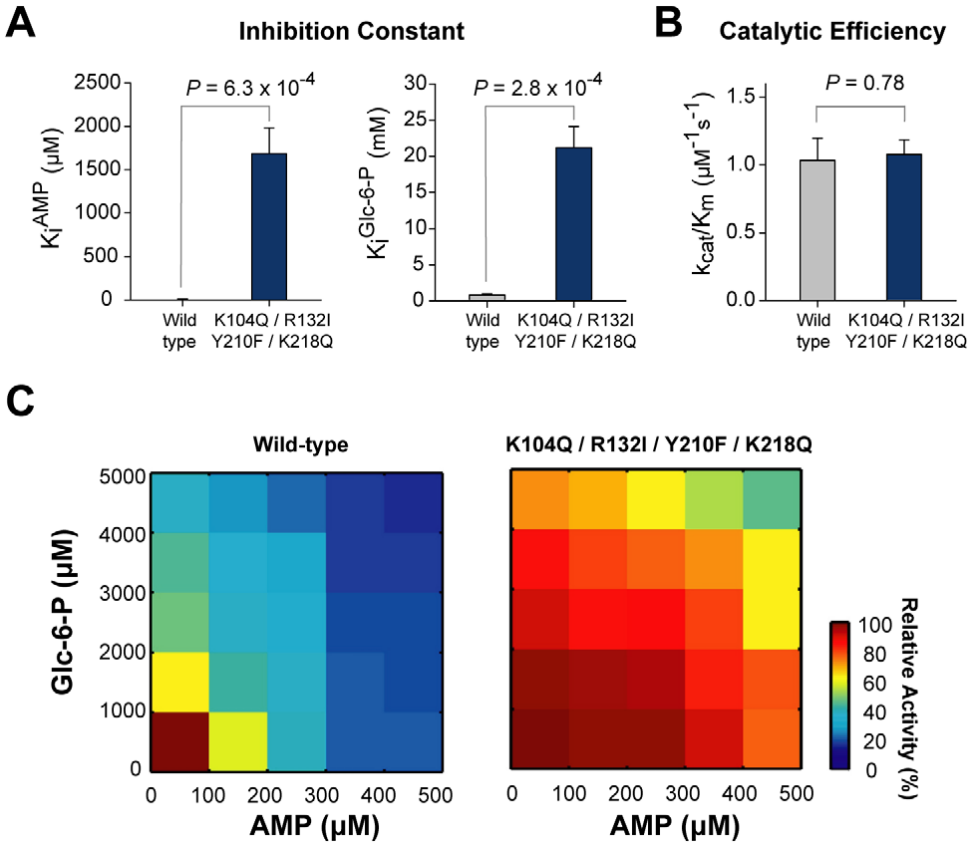
Combinatorial mutations of allosteric sites of *E. coli* FBPase. (A) Comparison of inhibition constants of wild-type and mutant FBPase by AMP and Glc-6-P. The statistical significance (*P*-value) was measured by t-test. (B) Comparison of catalytic efficiencies of wild-type and mutant FBPase. (C) The profile of catalytic activities of wild-type and mutant FBPase in the presence of various concentrations of AMP and Glc-6-P. The range of AMP concentrations was 0–500 µM and that of Glc-6-P was 0–5000 µM.

Next, we combined the four mutations (K104Q/R132I/Y210F/K218Q) to test whether they were effective in diminishing inhibition by both AMP and Glc-6-P simultaneously. The *K_i_* of the quadruple mutant was 170-fold higher for AMP (*P* = 6.3×10^−4^) and 25-fold higher for Glc-6-P (*P* = 2.8×10^−4^) than that of the wild type (Fig. 5*A* and Text S4). Additionally, as shown in Fig. 5*B*, the catalytic efficiency of the quadruple mutant was similar to that of the wild type (*P* = 0.78). Finally, we investigated the activity profiles of wild-type and the quadruple mutant FBPase by simultaneously changing AMP and Glc-6-P concentrations. At concentrations higher than 100 µM of AMP and 1000 µM of Glc-6-P, the relative activity of wild-type FBPase dramatically decreased to lower than 30% (Fig. 5*C*, left panel). However, the quadruple mutant FBPase was highly resistant to inhibition even in the presence of high concentrations of both AMP and Glc-6-P. Remarkably, the quadruple mutant FBPase retained >70% relative activity in the presence of 300 µM AMP and 3000 µM Glc-6-P (Fig. 5*C*, right panel). These results suggest that our mutation strategy succeeded in deregulating the allosteric inhibition of *E. coli* FBPase without impairing its catalytic efficiency.

### Mutations of the conserved residues in allosteric site diminished catalytic activity

We found that the mutations of conserved residues led to the loss of FBPase catalytic activity. We mutated the five conserved residues that interact with AMP or Glc-6-P and discovered that all the mutations had the complete loss of catalytic activity (Table 1). Two conserved residues in AMP binding site that are known to have favorable binding interactions with AMP via hydrogen bond (T23 and Y105) were selected [27], [28]. We mutated them into valine (T23V) and isoleucine (Y105I), respectively, to remove their hydrogen bonds, expecting that those mutations would lead to the loss of allosteric regulation only. In case of Glc-6-P binding sites, three conserved residues, Q225, E207, and Y203, which are corresponding to the formation of ionic and hydrogen bond with phosphate groups of Glc-6-P were selected. When we mutated these residues into isoleucine, leucine, or phenylalanine, all the mutations caused loss of catalytic function. These results indicate that conserved residues in allosteric sites are important for maintaining functional or structural constraint as well as binding allosteric regulator and propagating allosteric signal into catalytic site.

**Table 1:** Mutated residues of FBPase and the effect of mutations on allosteric inhibition and enzyme activity.

Allosteric Residues	Conservation Score	Mutations	Removing allosteric regulation without enzyme activity loss	Enzyme Activity	Allosteric Inhibition
**AMP binding pocket**					
**R132**	0.185	A, E, I	O	Sustained	Decreased
**K104**	0.384	Q	O	Sustained	Decreased
T23	0.547	V	X	Lost	Not detected
Y105	0.747	I	X	Lost	Not detected
**G6P binding pocket**					
**K222**	0.139	A, E, I, Q	O	Sustained	Decreased
**K218**	0.242	A, E, I, Q	O	Sustained	Decreased
**Y210**	0.399	F, I	O	Sustained	Decreased
Q225	0.605	I, L	X	Lost	Not detected
E207	0.65	I, L	X	Lost	Not detected
Y203	0.71	F	X	Lost	Not detected

Underlined characters represent mutated amino acids that led to the elimination of allosteric regulation without loss of enzyme activity. Residues with conservation scores<0.5 are highlighted with bold characters.

## Discussion

We analyzed the sequence evolution and amino acid compositions of catalytic and allosteric sites of enzymes. The evolution of ligand binding sites has been extensively investigated; however, catalytic and allosteric sites were not separately considered in many analyses of enzyme-ligand interactions [16]. Until recently, allosteric sites were expected to be conserved during the course of evolution, just as catalytic sites of enzymes are highly conserved to maintain their function [14]. However, several lines of evidence suggest that allosteric sites might be less conserved than catalytic sites, since allosteric regulation evidently evolved later than catalytic activity in enzymes along the course of evolution [29], [30]. Thus, different regulatory mechanisms may exist across species that result in sequence variations in regulatory sites [31]. Further, sequence variations at allosteric sites may be directly linked to the fine-tuning of regulation. Environmental conditions encountered by various species may dictate the necessity to alter the control of metabolic flux. Therefore, residues in allosteric sites would accordingly change from one species to another to allow variations in the specificity of allosteric regulators. Indeed, AMP does not activate yeast glycogen phosphorylase (GPb), but does activate vertebrate GPb, because the yeast enzyme lacks the residues that hydrogen bond with adenine [13].

Moreover, the physicochemical properties of catalytic and allosteric sites may differently affect their amino acid compositions. For instance, catalytic sites preferentially contain charged residues that help to stabilize the intermediate forms of substrates to promote bond formation or breakage [32]; these charged residues tend to be highly conserved across species to sustain the catalytic function of the enzyme. On the other hand, allosteric sites have larger numbers of hydrophobic residues to form binding pockets for allosteric ligands and a few charged residues for specific ligand interactions [33]. Therefore, amino acids in allosteric sites are more tolerant of mutations, because hydrophobic residues can be more easily replaced with similar sized residues compared to charged residues that are involved in specific interactions. We found that allosteric sites have more hydrophobic residues and less charged residues than catalytic sites. It might be possible few polar residues in allosteric sites might be crucial for the ligand specificity. Thus, those residues' changes have greater effect on the binding of polar ligands. For example, the replacement of polar amino acids to hydrophobic amino acids reduced the ligand binding in allosteric sites, which had been shown as increasing inhibition constants (*K_i_*). Although charged states of amino acids may be changed by the biochemical environment of enzymes, we note that sequence conservation analysis only takes into account the sequence variation or conservation of homologues. Environmental variations might already be reflected on the evolutionary constraints on functionally important sites.

The AMP binding site of *E.coli* FBPase is well characterized and several mutations which lead to the loss of allosteric inhibition are known. Therefore, we tried not to repeat the same mutations. In the AMP binding site, K104 and R132 are positively charged amino acids that directly contact with AMP [34] and these residues form hydrogen bonds with the phosphoryl groups of AMP. Thus, the mutations of these residues would disrupt their interactions that stabilize AMP binding. Meanwhile, Y210 and K222 provide large contact surfaces to Glc-6-P interaction (62.1 Å^2^ and 51.8 Å^2^, respectively) and form hydrogen bonds with the hydroxyl and phosphoryl groups in Glc-6-P [34]. Thus, the mutations of Y210 and K222 to other hydrophobic amino acids should perturb the interaction with Glc-6-P.

We searched for previous experimental data evaluating the effects of mutations in allosteric sites and compared with our analysis. We found that most successful allosteric deregulating mutations with no loss of catalytic activity correspond to residues that were less conserved (average conservation score = 0.47, Table S6), whereas mutations leading to a loss of catalytic activity correspond mostly to residues that were conserved (average conservation score = 0.88). These two group of residues were found to have significantly different conservation scores (*P* = 3.3×10^−5^; Mann-Whitney U test). However, we found some residues that did not follow the trend. For example, K42 and G191 in porcine FBPase are highly conserved but their mutation did not perturb the catalytic activity, whereas A54 is less conserved and its mutation perturbs the catalytic activity.

We provide the frequency of naturally occurring amino acids in Table S7. Amino acid substitutions that have successfully deregulated the allosteric control of enzyme were less frequently found in multiple sequence alignment (Average 3%). Because frequently occurring amino acids may work in allosteric ligand binding, we selected the less frequently occurring amino acids for the mutation experiments. Although allosteric site residues are more varied than active site residues, we found that allosteric sites are generally more conserved than surface residues. It has been suggested that allosteric sites are localized near protein-protein interfaces which are generally more conserved than surface residues [35]. Furthermore, residues in allosteric sites are also known to serve an important functional role in information propagation from the allosteric site to the active site. Allosteric sites are energetically connected with catalytic sites and coevolved during evolution [11]. These functional roles of allosteric sites might be one of the reasons that allosteric site residues are more conserved than surface residues.

Based on our sequence evolution analysis, we propose a novel engineering strategy to rationally modulate enzyme allosteric regulation. First, evolutionarily variable residues may be good targets for mutation because these residues tend to vary during evolution without losing a protein's activity. Mimicking natural evolution minimizes the probability of disrupting the catalytic activity of the enzyme [36]. In this study, we observed that all mutations in conserved residues invariably led to the loss of FBPase catalytic activity (Table 1), whereas mutations in variable residues generally did not result in loss of catalytic activity (0 out of 7 *versus* 10 out of 14, *P* = 3.8×10^−3^; Fisher's exact test). Second, amino acids that are likely to give selectivity by forming specific interactions with allosteric regulators via ionic or hydrogen bonds should be considered to mutate. Third, target residues should be substituted with less frequently occurring residues in nature, since frequently occurring residues might still play a role in allosteric regulation. We noted that further experimental validations are needed to establish the generality of our method. This residue selection strategy based on our evolutionary analysis, when combined with current protein engineering approaches, can facilitate the effective control of enzyme allosteric regulation. In addition, redesign of catalytic function would require the removal of the allosteric regulation of template enzymes to get rid of unwanted inhibition.

Understanding the evolutionary history of allosteric sites helped us to rationally design mutants for the allosteric control of FBPase. We successfully engineered an allosteric inhibition-resistant *E. coli* FBPase without impairing its catalytic efficiency. When *E. coli* is grown in minimal media containing glucose as a carbon source, intracellular concentrations of AMP and Glc-6-P are reported to reach concentrations of 280 µM and 2000 µM, respectively [37], [38]. Wild-type FBPase is inhibited to less than 20% by these inhibitor concentrations, but the quadruple mutant can maintain its enzyme activity at >80% of these inhibitor concentrations (Fig. 5*C*). In other words, the quadruple mutant FBPase engineered in this study can potentially enhance gluconeogenesis flux to regenerate reducing power (NADPH) through the pentose phosphate pathway even in the presence of elevated intracellular concentrations of AMP and Glc-6-P.

Furthermore, our results have implications on the identification of disease-causing mutations. Identification of disease-causing mutations from genome-wide association studies or next-generation sequencing studies currently focus on sequence conservation [39], which is based on the assumption that functionally important sites are conserved during evolution. Our findings support that mutations in allosteric sites may be responsible for deregulating enzyme allosteric control. Considering that dysfunction in allosteric regulation is highly associated with human disease, such as Alzheimer's disease and diabetes [40]–[42], our study provides a possible explanation of why mutation of evolutionary variable residues in allosteric sites can cause diseases. In fact, more than 20 disease-causing mutations in the allosteric regulator binding domain of pyruvate kinase are found to be evolutionarily less conserved [43], [44].

For the first time, we have systematically analyzed the evolutionary properties of enzyme allosteric sites. We found that residues in allosteric sites tend to be less conserved and more hydrophobic compared to those in highly conserved catalytic sites. Furthermore, we successfully deregulated the allosteric inhibition of FBPase without impairing its catalytic efficiency and propose a novel strategy for protein engineering. Recently, computational studies were shown to be quite powerful for identifying residues that deregulate allosteric behavior. For instance, a method combining molecular dynamic simulation and residue coevolution [11] was successfully applied to identify residues that are important for allosteric transition. Integrating such methods might improve the rational design of allosteric enzymes. We also expect that the sequence differences between allosteric and catalytic sites identified in this study will help to detect allosteric sites among potential ligand binding pockets, which currently relies on large-scale screening or serendipity [8], [35].

## Materials and Methods

### Catalytic, allosteric, and surface residues

We built two types of catalytic and allosteric site datasets. First, we constructed annotated catalytic and allosteric sites collected from hand-curated databases. The catalytic site atlas (CSA) contains experimentally confirmed catalytic sites and the allosteric database (ASD) has manually curated allosteric sites with at least three cases of experimental evidences in 3D structure or biochemistry [18], [19]. We found 56 allosteric proteins that have both catalytic and allosteric site annotations with solved 3D structures. Second, we constructed an alternative catalytic and allosteric site dataset. A hand-curated dataset contains high-quality data but might possess annotation bias or false negative residues. To overcome these limitations, we selected catalytic or allosteric residues from those within 6 Å of the substrates. This dataset contains more permissive residues with no annotation bias. Surface residues were defined as those that were highly solvent-accessible (>50% of relative solvent accessible surface area). NACCESS [45] was used to calculate solvent-accessible area.

### Sequence evolution analysis

We obtained the conservation scores and alignment files from the ConSurf server (http://consurfdb.tau.ac.il/) with default options. The server collected homologous sequences for calculating conservation scores from the UniProtKB/SwissProt database [46] using an *E*-value cutoff of 10^−3^ with three iterations of PSI-BLAST as previously described [47]. Then, it filtered out sequences with more than 95% identity to the query sequence and those that were shorter than 60% of the query sequence length. Lastly, redundant sequences were removed using CD-HIT [48]. Homologous sequences showed moderate sequence identities (36.9±12.6, Fig. S3). Conservation scores of residues were calculated from the resulting homologous sequence set using the Rate4Site algorithm [49]. We normalized the conservation scores by using the percentile normalization method to compare conservation scores of different enzymes. Using the same strategy, we collected homologous sequences of *E. coli* FBPase to calculate conservation scores. Also, we compared our results with other conservation score methods and found that results were similar. The results were statistically significant in the methods that we tested (from 10^−7^ to 10^−55^; Mann-Whitney U test, Table S8).

### Statistical analysis

We compared conservation scores between catalytic and allosteric sites in Fig. 1 and 3 based on Mann-Whitney U test which is suitable for assessing whether one of two samples have larger values than the other when data does not follow a normal distribution. We also measured the statistical significance (*P*-value) by Fisher's exact test to compare each amino acids composition in Fig. 2 with the null hypothesis as catalytic and allosteric sites have the same amino acid proportions. In addition, we performed t-test analysis to assess the difference of inhibition constants (*K_i_*) and catalytic efficiency (*k_cat_*/*K_m_*) respectively between wild type and mutants in Fig. 4 and 5. We obtained reproducible results from replicate experiments. A *P*<0.05 was considered to be statistically significant. All the statistical tests were done by scipy in python module.

### Reagents and primers

PrimeSTAR HS DNA polymerase and pET101/D-TOPO were purchased from Takara Bio Inc. (Shiga, Japan) and Invitrogen (Carlsbad, CA, USA), respectively. Oligonucleotides used for the construction of pET101/D-TOPO-FBPase and variants were synthesized by Bioneer (Daejeon, Korea). All other reagents were obtained from Sigma-Aldrich (St Louis, MO, USA).

### PCR-based site-directed mutagenesis

pET101/D-TOPO-FBPase was constructed by inserting the *fbp* gene, amplified from *E. coli* K-12 MG1655 genomic DNA using the FBP_CACC_F (CACCATGAAAACGTTAGGTGAATTTATTGTCGAAAAG) and FBP_B (CGCGTCCGGGAACTCACGGATAAA) primers, into pET101/D-TOPO following the manufacturer's instructions. This construct was then used as a template for amino acid substitutions by PCR-based site-directed mutagenesis. The PCR mixture for site-directed mutagenesis consisted of 50 ng pET101/D-TOPO-FBPase plasmid, 10 pmol of each primer, 1.25 U PrimeSTAR HS DNA polymerase, 250 µM of each dNTP, and 10 µl 5× buffer supplied by Takara Bio Inc. H_2_O was added to bring the final volume to 50 µl. PCR was carried out on an Applied Biosystems 2720 Thermal Cycler (Applied Biosystems, Foster City, CA, USA) under the following conditions: 98°C for 30 s, 12 cycles (point mutations) or 18 cycles (multiple nucleotide changes) of 98°C for 10 s, the appropriate primer T_m_-dependent annealing temperature for 15 s, and 68°C for 7 min, followed by a final extension at 68°C for 10 min. After thermocycling, the original template DNA was eliminated by treating with DpnI at 37°C for 1 h, and PCR products were isolated using a QIAquick PCR Purification Kit (Qiagen GmbH, Hilden, Germany). The *E. coli* Mach1™-T1R strain (Invitrogen) was transformed with 50 ng of the PCR product. Purified plasmids were sequenced by Solgent (Daejeon, Korea) using an ABI 3730XL capillary DNA Sequencer.

### Expression and purification of FBPases

The *E. coli* BL21 (DE3) strain (Invitrogen) was transformed with 50 ng of plasmid isolated and purified from the Mach1™-T1R strain, and clones containing the selected pET101/D-TOPO-FBPase and its variants were grown in LB medium containing 50 µg/ml ampicillin. After cultures had reached an OD_600_ of 0.4–0.6, determined using a UV-1700 spectrophotometer (Shimadzu, Kyoto, Japan), protein synthesis was induced by adding isopropyl-beta-D-thiogalactopyranoside (IPTG) to a final concentration of 1 mM. The induced cells were harvested by centrifugation at 4,000× *g* for 10 min at 4°C after incubating for an additional 4 h. Cells were resuspended in lysis buffer (50 mM HEPES, 300 mM NaCl, 10 mM imidazole, pH 8.0) containing lysozyme (Epicentre, Madison, WI, USA) and protease inhibitor (Sigma-Aldrich) and sonicated 30 times for 2 s each time with 8-s pauses in between, at a 20–30% duty cycle. Soluble lysates were separated by centrifugation at 10,000× *g* for 20 min at 4°C. FBPases with the polyhistidine-tag were purified by affinity chromatography using Ni-NTA agarose (Qiagen). Proteins attached to Ni-NTA agarose were washed twice with wash buffer I (50 mM HEPES, 300 mM NaCl, 20 mM imidazole, pH 8.0) and wash buffer II (50 mM HEPES, 300 mM NaCl, 40 mM imidazole, pH 8.0) and eluted with elution buffer (50 mM HEPES, 300 mM NaCl, 250 mM imidazole, pH 8.0). Eluted proteins were desalted using a PD-10 desalting column (GE Healthcare, Piscataway, NJ, USA). The concentrations of purified proteins were determined by Bio-Rad Protein Assay Kit (Bio-rad, Hercules, CA, USA) using BSA as a standard.

### Kinetic experiments

The activities of the purified FBPases were measured by monitoring the evolution of P_i_ from FBP using the Malachite Green Phosphate Assay Kit (BioAssay Systems, Hayward, CA, USA). Assay mixtures (50 mM HEPES, pH 7.5, 0.1 mM EDTA, 0.17 µg enzyme, and varying amounts of FBP, AMP, and Glc-6-P in a total volume of 80 µl) were incubated in microtiter plates (Oy Growth Curves Ab, Helsinki, Finland) at 25°C for 1 h prior to the initiation of the reaction by the addition of varying amounts of MgCl_2_. The addition of 20 µl of Working Reagent (BioAssay Systems) quenched the reactions, and the plates were incubated at 25°C for 30 min to allow color development. Absorbance (λ = 600 nm) was measured on Bioscreen C MBR (Oy Growth Curves Ab). A linear standard curve relating A_600_ to [P_i_] was drawn according to the manufacturer's guidelines (*R* ^2^>0.99).

Kinetic parameters such as *k_cat_*, *K_m_* (FBP, Mg^2+^), and the Hill coefficient were determined by fitting initial velocity data to Equation 1 (*R* ^2^>0.95),
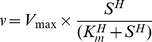
(1)where *v* is the velocity, *S* is either the concentration of FBP or Mg^2+^, *V_max_* is the velocity at saturating FBP and Mg^2+^, *K_m_* is the Michaelis constant for either FBP or Mg^2+^, and *H* is the Hill coefficient.

Inhibition data by AMP and Glc-6-P with respect to FBP were fit to the following equation (Equation 2) for nonlinear noncompetitive inhibition (*R* ^2^>0.95),
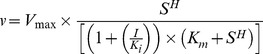
(2)where *v* is the velocity, *V_max_* is the velocity with saturating FBP and Mg^2+^ with no inhibitor present, *K_m_* is the Michaelis constant for FBP, *S* is the concentration of FBP, *K_i_* is the inhibition constant, *I* is the concentration of either AMP or Glc-6-P, and *H* is the Hill coefficient for FBP derived from Equation 1. All data fitting were performed using GraphPad Prism 5.0 software (GraphPad Software Inc., La Jolla, CA, USA). All kinetic parameters measured in this study are listed in Table S5.

## Supporting Information

Figure S1 **Differences in the evolutionary and physicochemical properties of catalytic and allosteric sites.** (A) Distribution of conservation scores from catalytic and allosteric sites of enzymes. Catalytic site (522 residues) and allosteric site (782 residues) residues were defined as amino acid residues within 6 Å of the substrate. The statistical significance (*P*-value) was measured by the Mann-Whitney U test. (B) Amino acid proportion of catalytic and allosteric site residues. Fraction of each amino acid of catalytic and allosteric residues is shown. Catalytic site (522 residues) and allosteric site (782 residues) residues were defined as amino acid residues within 6 Å of the substrate. Allosteric sites have more hydrophobic residues compared to catalytic sites, while catalytic sites have more charged amino acid than do allosteric sites. The statistical significance (*P*-value) was measured by Fisher's exact test; *P<0.05 and **P<0.005.
(TIF)

Figure S2 **Relative activities of wild-type and mutant FBPase in the presence of AMP and Glc-6-P.** (A) Catalytic efficiency of wild-type FBPase and AMP binding site mutants in the presence of AMP. (B) Catalytic efficiency of wild-type FBPase and AMP binding site mutants in the presence of Glc-6-P. (C) Catalytic efficiency of wild-type FBPase and Glc-6-P binding site mutants in the presence of AMP. (D) Catalytic efficiency of wild-type FBPase and Glc-6-P binding site mutants in the presence of Glc-6-P. (E) Catalytic efficiency of wild-type FBPase and AMP and Glc-6-P binding site mutants in the presence of AMP. (F) Catalytic efficiency of wild-type FBPase and AMP and Glc-6-P binding site mutants in the presence of Glc-6-P.
(TIF)

Figure S3 **Distributions of sequence identity (in percentage) calculated from multiple alignments of homologous sequences.** The distributions of sequence identities of 56 allosteric proteins are shown. We collected sequence identities from multiple sequence alignment by comparing a query protein and its homologous sequences. Overall, homologous sequences showed moderate sequence identities (36.9±12.6) compared to their query sequences.
(TIF)

Figure S4 **Distribution of conservation scores from catalytic and allosteric sites in aspartate carbamoyltransferase (ATCase) and glycogen phosphorylase (GPb).** The structures of ATCase (upper, left; PDB code 2FZC) and GPb (lower, left; PDB code 7GPB) are shown. Distribution of conservation scores from catalytic, allosteric, and surface residues. The residue annotation was constructed from the distance from the substrates. The statistical significance (*P*-value) was measured by the Mann-Whitney U test.
(TIF)

Table S1 **Conservation scores of catalytic and allosteric sites of 56 allosteric proteins.**
(XLS)

Table S2 **Distributions of enzyme classes in our dataset and the entire ENZYME database.**
(XLS)

Table S3 **Composition of amino acids in catalytic and allosteric sites of 56 allosteric proteins.**
(XLS)

Table S4 **Conservation scores of catalytic, allosteric, and surface residues of ATCase, GPb, and FBPase.**
(XLS)

Table S5 **Kinetic parameters of FBPase and its mutants.**
(XLS)

Table S6 **Kinetic parameters and conservation scores of mutation sites with experimental references.**
(XLS)

Table S7 **Amino acid substitutions at the engineered sites found in homologous sequences.**
(XLS)

Table S8 **Conservation scores of the catalytic and allosteric sites of 56 enzymes by other sequence analysis methods.**
(XLS)

Table S9 **Conservation scores of catalytic, allosteric, and surface residues of ATCase, GPb, and FBPase calculated by using ortholog sequences.**
(XLS)

Text S1 **Allosteric sites are found to be less conserved than catalytic sites in each enzyme.**
(DOC)

Text S2 **Mutations in less conserved residues diminish the inhibitory effect of AMP.**
(DOC)

Text S3 **Mutations in less conserved residues diminish the inhibitory effect of Glc-6-P.**
(DOC)

Text S4 **Mutant FBPase resistant to inhibition by both AMP and Glc-6-P.**
(DOC)
