# Supplementary figures and images for: Rational Engineering of Enzyme Allosteric Regulation through Sequence Evolution Analysis
Source: PLoS Comput Biol. 2012 Jul 12;8(7):e1002612. doi: 10.1371/journal.pcbi.1002612 (PMC3395594; doi:10.1371/journal.pcbi.1002612)

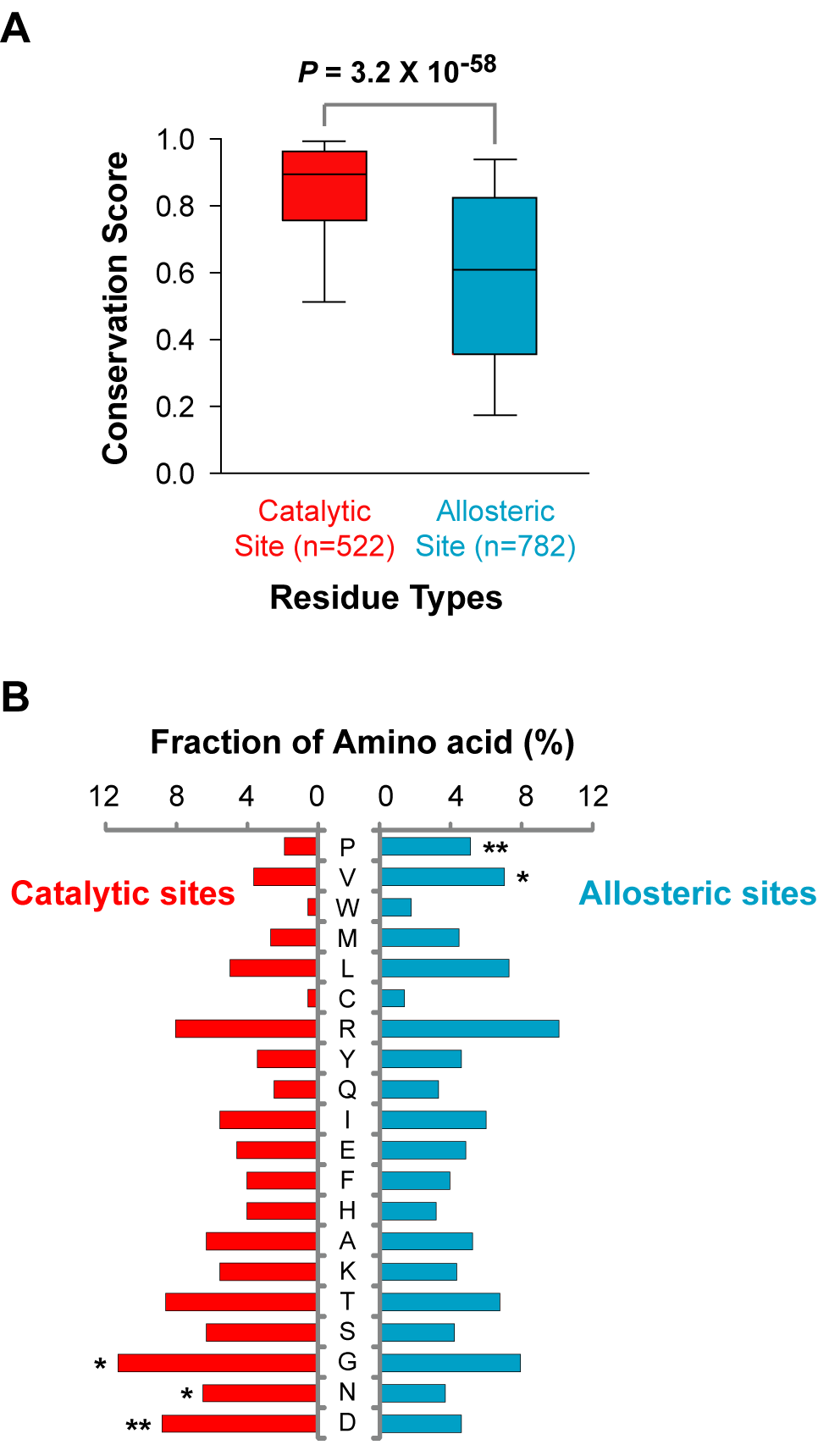

Supplement: Figure S1 — Differences in the evolutionary and physicochemical properties of catalytic and allosteric sites. (A) Distribution of conservation scores from catalytic and allosteric sites of enzymes. Catalytic site (522 residues) and allosteric site (782 residues) residues were defined as amino acid residues within 6 Å of the substrate. The statistical significance (P-value) was measured by the Mann-Whitney U test. (B) Amino acid proportion of catalytic and allosteric site residues. Fraction of each amino acid of catalytic and allosteric residues is shown. Catalytic site (522 residues) and allosteric site (782 residues) residues were defined as amino acid residues within 6 Å of the substrate. Allosteric sites have more hydrophobic residues compared to catalytic sites, while catalytic sites have more charged amino acid than do allosteric sites. The statistical significance (P-value) was measured by Fisher's exact test; *P<0.05 and **P<0.005. (TIF) [file pcbi.1002612.s001.tif]

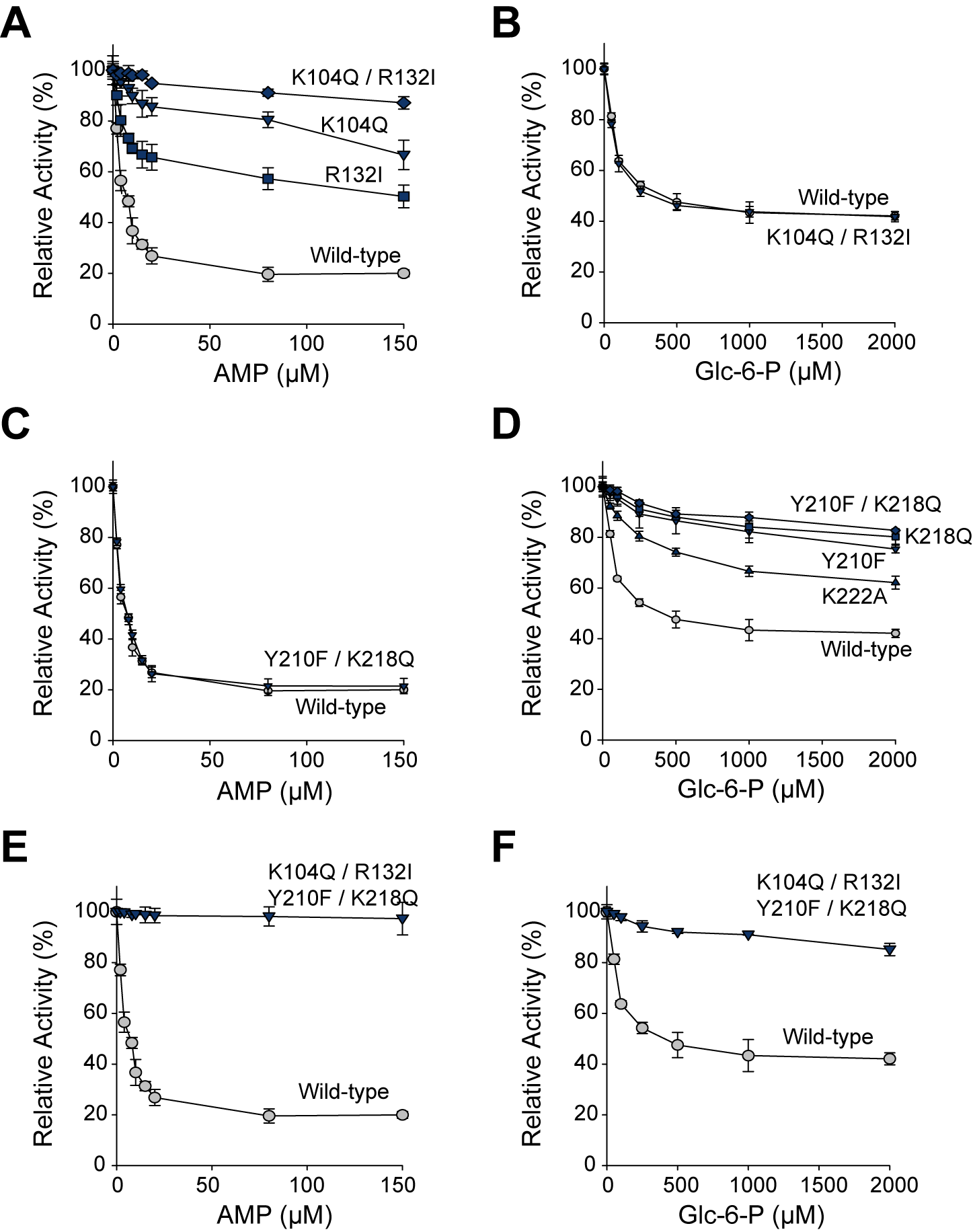

Supplement: Figure S2 — Relative activities of wild-type and mutant FBPase in the presence of AMP and Glc-6-P. (A) Catalytic efficiency of wild-type FBPase and AMP binding site mutants in the presence of AMP. (B) Catalytic efficiency of wild-type FBPase and AMP binding site mutants in the presence of Glc-6-P. (C) Catalytic efficiency of wild-type FBPase and Glc-6-P binding site mutants in the presence of AMP. (D) Catalytic efficiency of wild-type FBPase and Glc-6-P binding site mutants in the presence of Glc-6-P. (E) Catalytic efficiency of wild-type FBPase and AMP and Glc-6-P binding site mutants in the presence of AMP. (F) Catalytic efficiency of wild-type FBPase and AMP and Glc-6-P binding site mutants in the presence of Glc-6-P. (TIF) [file pcbi.1002612.s002.tif]

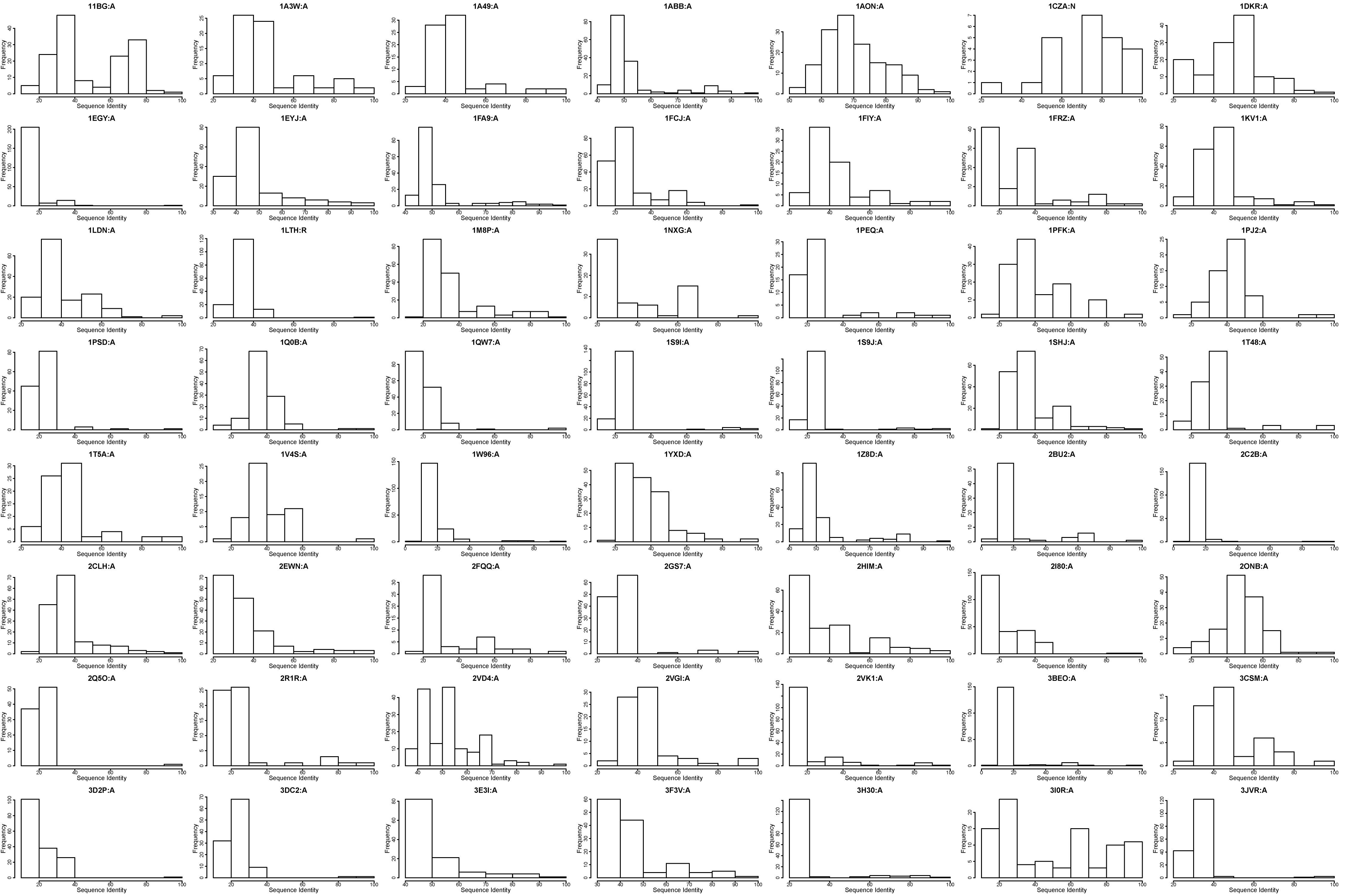

Supplement: Figure S3 — Distributions of sequence identity (in percentage) calculated from multiple alignments of homologous sequences. The distributions of sequence identities of 56 allosteric proteins are shown. We collected sequence identities from multiple sequence alignment by comparing a query protein and its homologous sequences. Overall, homologous sequences showed moderate sequence identities (36.9±12.6) compared to their query sequences. (TIF) [file pcbi.1002612.s003.tif]

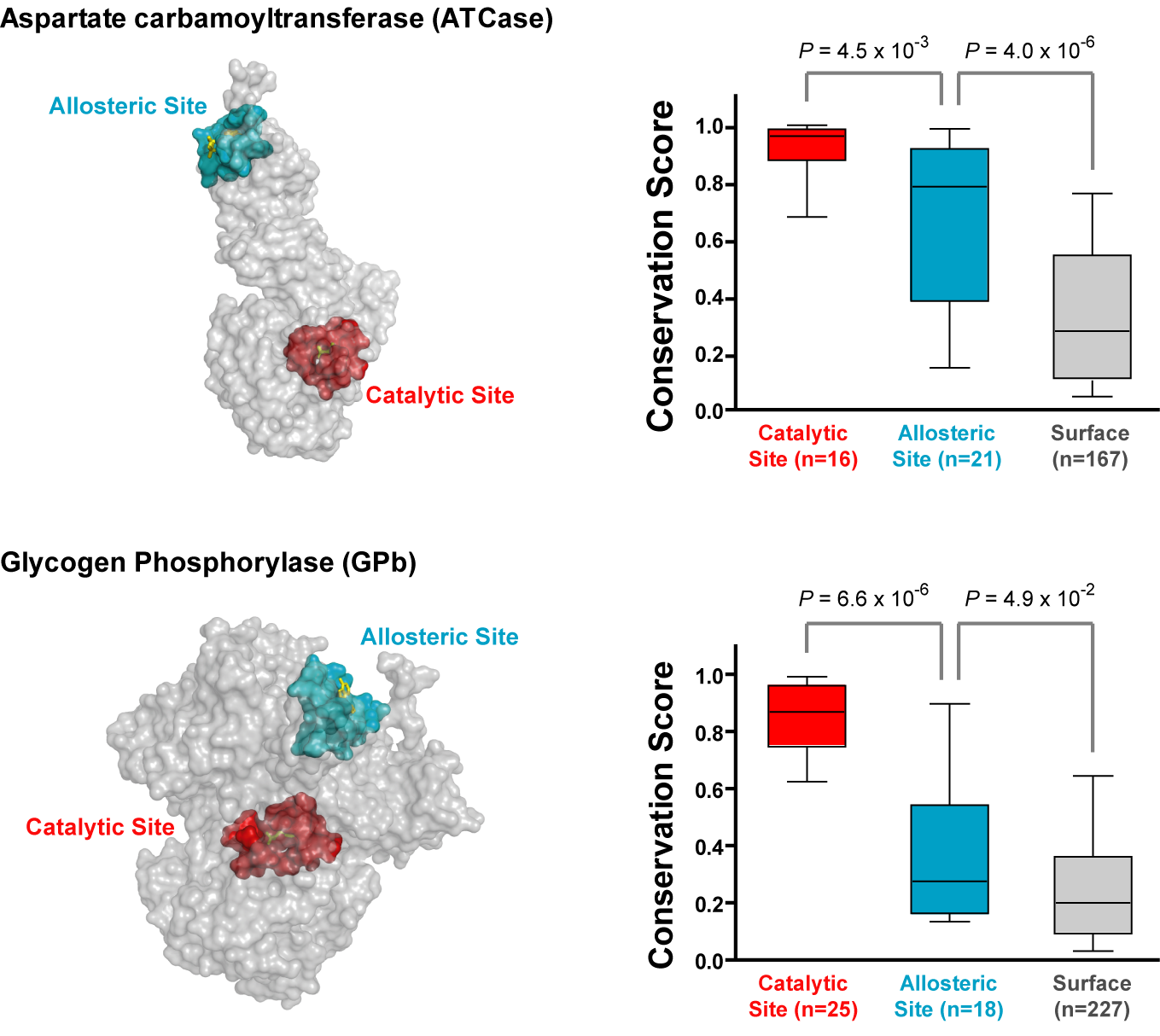

Supplement: Figure S4 — Distribution of conservation scores from catalytic and allosteric sites in aspartate carbamoyltransferase (ATCase) and glycogen phosphorylase (GPb). The structures of ATCase (upper, left; PDB code 2FZC) and GPb (lower, left; PDB code 7GPB) are shown. Distribution of conservation scores from catalytic, allosteric, and surface residues. The residue annotation was constructed from the distance from the substrates. The statistical significance (P-value) was measured by the Mann-Whitney U test. (TIF) [file pcbi.1002612.s004.tif]
