# Supplementary material for: Rational Engineering of Enzyme Allosteric Regulation through Sequence Evolution Analysis
Source: PLoS Comput Biol. 2012 Jul 12;8(7):e1002612. doi: 10.1371/journal.pcbi.1002612 (PMC3395594; doi:10.1371/journal.pcbi.1002612)
Supplement: Text S1 — Allosteric sites are found to be less conserved than catalytic sites in each enzyme. (DOC) [file pcbi.1002612.s014.doc]

**Text S1. Allosteric sites are found to be less conserved than catalytic sites in each enzyme.**

We observed that conservation tendencies were the same in an individual protein. For example, aspartate carbamoyltransferase (ATCase), a key enzyme that catalyzes the first step in pyrimidine biosynthesis, consists of two different catalytic and regulatory subunits. Binding of CTP to the allosteric site in the regulatory subunit results in an equilibrium shift towards the tense state, which in turn leads to low activity and low affinity to substrate. When we calculated conservation scores separately in each subunit, we found that residues in the allosteric site were less conserved than those in the catalytic site in ATCase (*P* = 4.5 × 10^-3^, Fig. S4, Table S4). Another example is glycogen phosphorylase (GPb), which catalyzes the rate-limiting step in the degradation of glycogen in animals. This enzyme is regulated by allosteric control through the binding of AMP. An increase in AMP concentration during exercise activates GPb by changing its conformation from the tense to the relaxed form. As seen in the case of ATCase, residues in the allosteric activator binding site of GPb are less conserved than those in the catalytic site (*P* = 6.6 × 10^-6^, Fig. S4, Table S4).

Our conservation analysis was based on homologous sequences, so functionally analogous enzyme sets might be considered. To minimize these analogues effect, we compiled only functional ortholog genes by using swissprot annotation. With these sequence sets, we compared the sequence conservation among catalytic, allosteric and surface residues of three representative enzymes, FBPase, ATCase, and GPb (Table S9). We found that our conclusion is still robust with ortholog gene sets in FBPase and ATCase. But, we could not distinguish catalytic, allosteric and surface residues with conservation scores in our muscle form GPb which belong to GPb family. The ortholog sequences of muscle form GPb were too small and similar with each other to calculate conservation (7 sequences and average sequence identities = 90%).
