# Supplementary material for: Rational Engineering of Enzyme Allosteric Regulation through Sequence Evolution Analysis
Source: PLoS Comput Biol. 2012 Jul 12;8(7):e1002612. doi: 10.1371/journal.pcbi.1002612 (PMC3395594; doi:10.1371/journal.pcbi.1002612)
Supplement: Text S2 — Mutations in less conserved residues diminish the inhibitory effect of AMP. (DOC) [file pcbi.1002612.s015.doc]

**Text S2. Mutations in less conserved residues diminish the inhibitory effect of AMP**

First of all, the relative activities of mutants as a function of AMP concentration (0–150 μM) were investigated. The R132I and K104Q mutations resulted in 60% and 70% relative activity of FBPase, respectively, though that of the wild type was reduced to 20%, in the presence of AMP (Fig. S2*A*). In addition, a double mutant combining these two mutations (K104Q/R132I) was much more resistant to AMP inhibition, with a relative activity of over 90% (Fig. S2*A*).
