# Supplementary material for: Rational Engineering of Enzyme Allosteric Regulation through Sequence Evolution Analysis
Source: PLoS Comput Biol. 2012 Jul 12;8(7):e1002612. doi: 10.1371/journal.pcbi.1002612 (PMC3395594; doi:10.1371/journal.pcbi.1002612)
Supplement: Text S3 — Mutations in less conserved residues diminish the inhibitory effect of Glc-6-P. (DOC) [file pcbi.1002612.s016.doc]

**Text S3. Mutations in less conserved residues diminish the inhibitory effect of Glc-6-P**

The relative activities of K218Q and Y210F mutants were >80% as the concentration of Glc-6-P approached 2000 μM (Fig. S2*D*). On the other hand, the relative activities of K222A and wild-type FBPase were decreased to about 60% and 40%, respectively. To further improve the resistance to Glc-6-P inhibition, Y210F and K218Q, which were each highly effective in deregulation, were combined; the double mutant (Y210F/K218Q) retained >90% relative activity (Fig. S2*D*).
