# Supplementary material for: Rational Engineering of Enzyme Allosteric Regulation through Sequence Evolution Analysis
Source: PLoS Comput Biol. 2012 Jul 12;8(7):e1002612. doi: 10.1371/journal.pcbi.1002612 (PMC3395594; doi:10.1371/journal.pcbi.1002612)
Supplement: Text S4 — Mutant FBPase resistant to inhibition by both AMP and Glc-6-P. (DOC) [file pcbi.1002612.s017.doc]

**Text S4. Mutant FBPase resistant to inhibition by both AMP and Glc-6-P**

The quadruple mutant FBPase (K104Q/R132I/Y210F/K218Q) retained >90% relative activity with increasing concentrations of AMP and Glc-6P (AMP, 0–150 μM; Glc-6-P, 0–2000 μM; Fig. S2*E* and *F*).
